# Prognostic value of the triglyceride–glucose index in patients with premature acute coronary syndrome

**DOI:** 10.1186/s12872-026-06263-0

**Published:** 2026-07-20

**Authors:** Yifan Sun, Jibin Chen, Nianzu Wei, Ming Zhang, Chunquan Zhang, Wei Xia, Lei Zhang, Yibing Shao, Bin Wang

**Affiliations:** 1 Shandong Second Medical University, Weifang, China; 2 Department of Cardiology, Qingdao Municipal Hospital, University of Health and Rehabilitation Sciences, Qingdao, China

**Keywords:** Premature acute coronary syndrome, Triglyceride-glucose index, Major adverse cardiovascular and cerebrovascular events, Prognosis, Insulin resistance

## Abstract

**Background:**

The triglyceride-glucose (TyG) index has been proposed as an accessible indicator of insulin resistance and cardiometabolic risk. However, its prognostic relevance in patients with premature acute coronary syndrome has not been well defined. We therefore examined baseline clinical features and long-term cardiovascular outcomes in patients with premature acute coronary syndrome (PACS) stratified by TyG status.

**Methods:**

This retrospective cohort study screened 820 consecutive patients hospitalized with PACS, of whom 804 had complete fasting triglyceride and fasting plasma glucose measurements for TyG calculation. TyG was calculated as ln [fasting triglycerides (mg/dL) × fasting plasma glucose (mg/dL) / 2]. Patients were categorized into high and low TyG groups using the 66.7th percentile as the cutoff. The primary outcome was major adverse cardiovascular and cerebrovascular events (MACCE).

**Results:**

Among 804 eligible patients, 268 (33.3%) were assigned to the high TyG group and 536 (66.7%) to the low TyG group. Patients with high TyG showed a more unfavorable cardiometabolic profile and a higher coronary disease burden at baseline than those with low TyG. During follow-up, MACCE occurred in 17.2% of patients in the high TyG group and in 10.4% of those in the low TyG group. After multivariable adjustment, high TyG remained associated with a higher risk of MACCE (adjusted HR 1.61, 95% CI 1.05–2.47; *P* = 0.029). TyG analyzed as a standardized continuous variable was also independently associated with MACCE (adjusted HR 1.306, 95% CI 1.039–1.643; *P* = 0.022). Landmark analysis indicated that the association was more prominent after 365 days.

**Conclusion:**

In this PACS cohort, elevated TyG was independently associated with an increased risk of MACCE, particularly during longer-term follow-up. TyG may provide a simple adjunctive marker for identifying residual cardiometabolic risk in relatively young patients after ACS.

## Introduction

Premature acute coronary syndrome (PACS) represents an important clinical phenotype of coronary artery disease because the index event occurs early in life and may be followed by decades of residual cardiovascular risk [1]. Although contemporary diagnostic pathways, revascularization strategies, and secondary prevention have improved the management of acute coronary syndrome (ACS), ACS continues to contribute substantially to global cardiovascular morbidity and mortality [2]. Registry data suggest that younger patients with myocardial infarction frequently present with modifiable cardiometabolic risk factors, making refined risk assessment particularly relevant in this population [3]. In addition, recent multicenter evidence indicates that premature acute myocardial infarction is not necessarily benign and that multivessel disease may confer a sustained adverse prognostic effect despite the younger age and lower comorbidity burden of these patients [4]. Therefore, PACS should not be regarded simply as ACS occurring at a younger age, but rather as a distinct clinical scenario characterized by persistent residual cardiovascular risk. This feature makes simple and clinically available markers for long-term risk stratification particularly relevant in this population.

The triglyceride-glucose (TyG) index combines fasting triglyceride and fasting glucose concentrations and has been widely used as a practical non-insulin-based proxy for insulin resistance [5]. Its validity is supported by comparisons with the hyperinsulinemic-euglycemic clamp, a reference method for assessing insulin sensitivity [6]. From a pathophysiological perspective, insulin resistance and lipotoxicity may accelerate atherosclerosis through endothelial dysfunction, oxidative stress, inflammatory activation, and adverse vascular remodeling [7]. Therefore, TyG may capture an integrated metabolic disturbance that is not fully reflected by glucose or lipid variables considered separately, although the marker also has recognized limitations in clinical interpretation [8].

Accumulating evidence has linked higher TyG values with adverse outcomes in ACS and post-percutaneous coronary intervention (PCI) settings. A dose-response meta-analysis reported an association between TyG and major adverse cardiovascular events in ACS [9]. Similar findings have been described among patients with diabetes and ACS [10], patients with type 2 diabetes mellitus undergoing PCI for ACS [11], and nondiabetic patients with non-ST-segment elevation ACS treated with elective PCI [12]. TyG has also been reported to add prognostic information to established clinical risk models after ACS [13]. Beyond a single baseline measurement, both TyG level and TyG variability after PCI have been associated with subsequent outcomes [14]. More recent cohorts have extended these observations to nondiabetic ACS patients undergoing emergency PCI with drug-eluting stents [15] and to patients undergoing complex coronary intervention [16], while pooled post-PCI evidence has further supported the relationship between elevated TyG and worse cardiovascular prognosis [17].

Nevertheless, the evidence base remains limited for PACS specifically. This distinction matters because younger ACS patients may experience a longer duration of exposure to insulin resistance and related metabolic abnormalities after the index event, potentially modifying the timing and magnitude of long-term risk. Accordingly, the present study was designed to describe the clinical and angiographic profile of patients with PACS according to TyG status and to evaluate whether TyG is associated with subsequent cardiovascular events during follow-up.

### Study design and participants

This retrospective cohort study included patients hospitalized for premature acute coronary syndrome at Qingdao Municipal Hospital, University of Health and Rehabilitation Sciences (Qingdao, China) from January 2019 to January 2020, with outcome follow-up continued through December 2023. Eligible diagnoses included ST-segment elevation myocardial infarction (STEMI) and non-ST-segment elevation acute coronary syndrome (NSTE-ACS). STEMI was identified by ischemic symptoms accompanied by persistent ST-segment elevation on electrocardiography. NSTE-ACS comprised non-ST-segment elevation myocardial infarction (NSTEMI) and unstable angina (UA). NSTEMI was defined by ongoing ischemic symptoms and elevated cardiac troponin without ST-segment elevation, whereas UA was defined by symptoms consistent with myocardial ischemia in the absence of troponin elevation. PACS was defined as disease onset at ≤ 55 years in men or ≤ 65 years in women.

The study was prepared in accordance with the Strengthening the Reporting of Observational Studies in Epidemiology (STROBE) statement [18] and conducted in line with the Declaration of Helsinki [19]. Clinical care was interpreted with reference to contemporary guideline-directed recommendations for ACS management [20]. The study protocol was reviewed and approved by the Ethics Committee of Qingdao Municipal Hospital, University of Health and Rehabilitation Sciences (approval number: 2023-KY-074). Written informed consent was obtained from all participants.

**Fig. 1:**
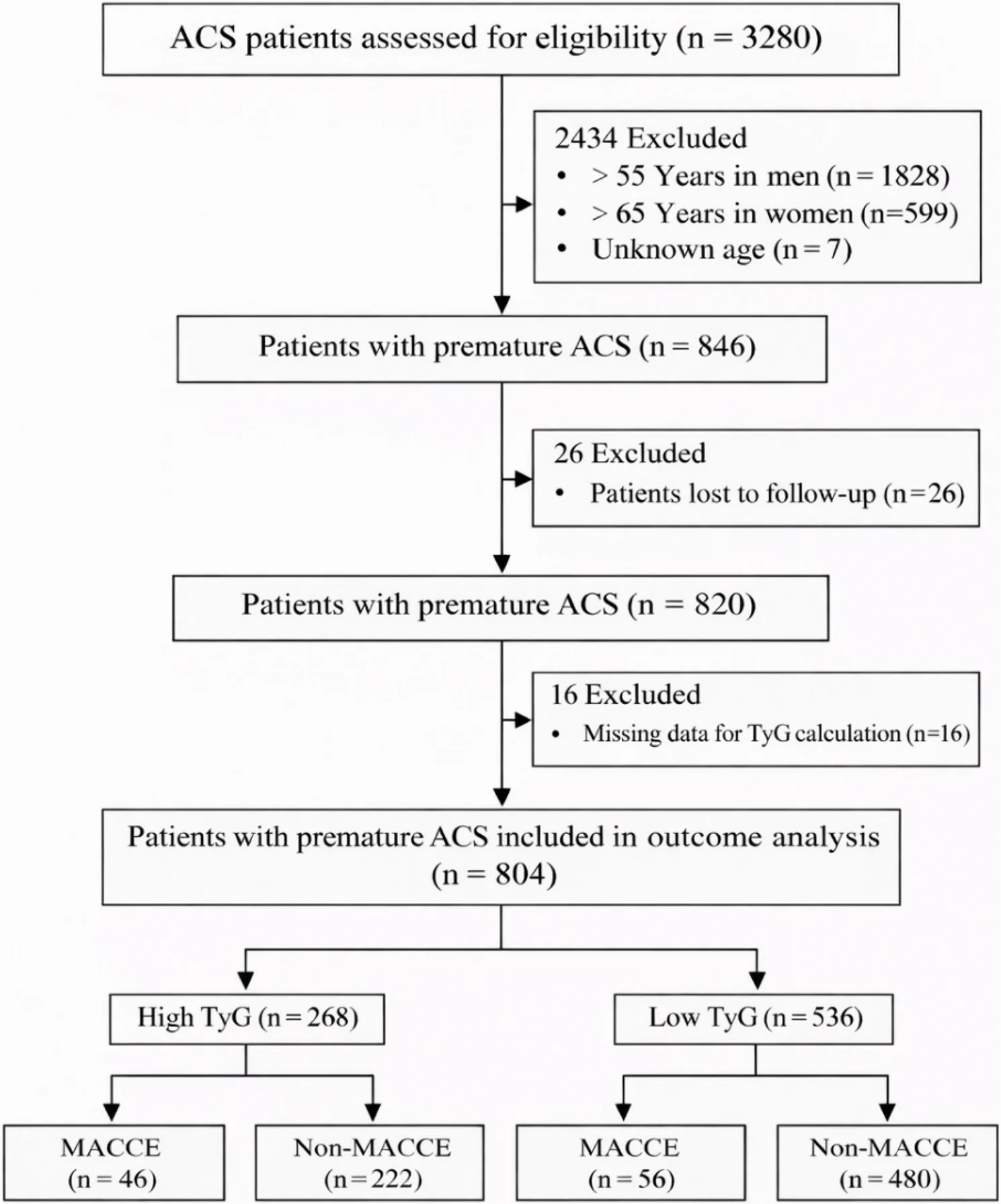
Study flowchart. ACS: acute coronary syndrome; MACCE: major adverse cardiovascular and cerebrovascular event

Of 820 screened patients with PACS, 804 had the fasting triglyceride and fasting plasma glucose data required to calculate TyG and were included in the complete-case analysis. Patients lacking variables necessary for TyG calculation were excluded (Fig. 1).

### Procedures and management

TyG was calculated using the formula ln [fasting triglycerides (mg/dL) × fasting plasma glucose (mg/dL) / 2]. The 66.7th percentile of TyG was used to define the high TyG group, and the remaining patients were categorized as the low TyG group. Because no universally accepted TyG cutoff has been established for patients with PACS, this threshold was selected to identify patients with a distinctly elevated TyG burden while preserving adequate sample size and event numbers in both comparison groups. To evaluate whether the observed association was dependent on this categorization strategy, a median-based dichotomization was additionally performed as a sensitivity analysis. During the index ACS hospitalization, patients received standard medical therapy according to their clinical condition, including aspirin, clopidogrel or ticagrelor, low-molecular-weight heparin, statins, isosorbide mononitrate, and angiotensin-converting enzyme inhibitors/angiotensin II receptor blockers or beta-blockers as appropriate for heart rate, blood pressure, and individual contraindications. PCI was performed by experienced senior interventional cardiologists, and angiographic lesion characteristics were assessed visually. Discharge secondary prevention included dual antiplatelet therapy, beta-blockers, statins, angiotensin-converting enzyme inhibitors or angiotensin receptor blockers, smoking cessation counseling, and cardiac rehabilitation counseling when clinically indicated.

### Follow-up and end-points

After discharge, patients were followed through outpatient visits, medical record review, or telephone interviews conducted by research staff blinded to baseline clinical characteristics. Follow-up time was defined from discharge to the first endpoint event or, for patients without an event, to the last available follow-up date.

The primary endpoint was MACCE, a composite of cardiovascular death, myocardial infarction, stroke, ischemia-driven revascularization, or hospitalization for unstable angina or heart failure.

### Statistical analyses

Time-to-event outcomes were evaluated using Kaplan-Meier curves and compared with log-rank tests. Cox proportional hazards models were used to estimate hazard ratios (HRs) and 95% confidence intervals (CIs) in univariable and multivariable analyses. Adjustment variables were selected a priori according to clinical relevance and potential confounding in cardiovascular prognosis, including age, sex, body mass index, hypertension, diabetes mellitus, dyslipidemia, prior myocardial infarction, prior stroke, multivessel disease, clinical presentation, revascularization, and smoking status.

The proportional hazards assumption was examined because, when this assumption is not satisfied, a conventional Cox estimate should be interpreted as an average association across follow-up rather than a constant effect at every time point [21]. This assumption was assessed by adding a time-dependent interaction term between TyG and ln(time + 1). Given evidence suggesting a time-varying association for TyG, additional temporal analyses were performed. A landmark analysis using 365 days as the cutoff was performed because 1 year is a clinically meaningful interval in cardiovascular outcome studies and avoids selecting a cutoff solely from the observed data [22]. TyG was also analyzed as a standardized continuous variable (ZTyG). As a sensitivity analysis, patients were also dichotomized according to the median TyG value into higher and lower one-half groups to evaluate whether the association was consistent using an alternative cutoff. Restricted cubic spline analysis was used to characterize the dose-response pattern between TyG and MACCE and to evaluate potential nonlinearity without relying only on a categorical exposure definition [23]. Two-sided P values < 0.05 were considered statistically significant. Statistical analyses were performed using R software version 4.5.1, with restricted cubic spline analyses conducted using the rms package.

### Baseline clinical and procedural characteristics

The final cohort consisted of 804 patients, including 268 (33.3%) in the high TyG group and 536 (66.7%) in the low TyG group. Compared with patients in the low TyG group, those in the high TyG group more frequently had diabetes mellitus (50.0% vs. 19.4%, *P* < 0.001), hypertension (71.3% vs. 59.9%, *P* = 0.002), and dyslipidemia (45.1% vs. 30.2%, *P* < 0.001). The high TyG group also had higher body mass index, waist circumference, waist-to-hip ratio, diastolic blood pressure, fasting blood glucose, hemoglobin A1c, triglycerides, total cholesterol, and high-sensitivity C-reactive protein, together with lower high-density lipoprotein cholesterol. These baseline differences indicate a more adverse cardiometabolic phenotype among patients with elevated TyG.

Angiographic and procedural findings also differed by TyG status. Right coronary artery involvement and PCI use were more frequent in the high TyG group, and the number of diseased vessels was greater. By contrast, left main, left anterior descending, and left circumflex artery involvement did not differ significantly between groups. Overall, elevated TyG was accompanied by a greater coronary lesion burden and more frequent revascularization (Table 1). 

**Table 1:** Baseline clinical and procedural characteristics according to TyG group

Characteristic	Overall(n=804)	**Low TyG** **(n=536)**	**High TyG** **(n=268)**	***P*** ** value**
Demographics				
Age, years	50.0 (45.0–54.0)	51.0 (46.0–54.0)	49.0 (43.0–54.0)	0.003
Sex, male/female, n (%)	647/157 (80.5/19.5)	432/104 (80.6/19.4)	215/53 (80.2/19.8)	0.900
BMI, kg/m²	27.3 (24.9–29.8)	26.7 (24.3–29.4)	28.3 (26.0–30.4)	<0.001
Waist, cm	100.0 (94.0–106.0)	98.0 (92.0–104.0)	102.0 (96.0–108.0)	<0.001
Waist-to-hip ratio	1.0 (0.9–1.0)	1.0 (0.9–1.0)	1.0 (1.0–1.0)	<0.001
Systolic BP, mm Hg	125.0 (116.0–137.0)	125.0 (116.0–136.0)	126.0 (117.0–138.0)	0.149
Diastolic BP, mm Hg	78.0 (70.0–85.2)	76.0 (70.0–84.0)	80.0 (70.0–88.0)	0.001
Medical history				
Diabetes mellitus	238 (29.6%)	104 (19.4%)	134 (50.0%)	<0.001
Hypertension	512 (63.7%)	321 (59.9%)	191 (71.3%)	0.002
Dyslipidemia	283 (35.2%)	162 (30.2%)	121 (45.1%)	<0.001
Family history of PCAD	47 (5.8%)	32 (6.0%)	15 (5.6%)	0.832
Prior myocardial infarction	118 (14.7%)	82 (15.3%)	36 (13.4%)	0.481
Prior stroke	57 (7.1%)	37 (6.9%)	20 (7.5%)	0.771
Previous PCI	159 (19.8%)	101 (18.8%)	58 (21.6%)	0.348
Previous CABG	8 (1.0%)	4 (0.7%)	4 (1.5%)	0.452
Smoking	276 (34.3%)	187 (34.9%)	89 (33.2%)	0.636
Drinking	494 (61.4%)	347 (64.7%)	147 (54.9%)	0.007
Baseline tests				
Glucose, mmol/L	5.9 (5.3–7.5)	5.6 (5.1–6.3)	7.8 (6.0–10.9)	<0.001
Hemoglobin A1C, %	6.0 (5.5–6.9)	5.8 (5.4–6.3)	7.0 (5.9–8.6)	<0.001
Triglyceride, mmol/L	1.6 (1.1–2.2)	1.3 (1.0–1.6)	2.6 (2.0–3.3)	<0.001
Total Cholesterol, mmol/L	4.1 (3.4–4.9)	4.0 (3.3–4.7)	4.4 (3.8–5.2)	<0.001
HDL-C, mmol/L	1.0 (0.9–1.1)	1.0 (0.9–1.2)	0.9 (0.8–1.0)	<0.001
LDL-C, mmol/L	2.4 (1.9–3.2)	2.4 (1.9–3.2)	2.6 (2.0–3.2)	0.173
Hs-CRP, mg/L	2.1 (0.8–6.3)	1.9 (0.7–6.0)	2.6 (1.1–7.9)	0.003
LVEF, %	62.0 (58.0–66.0)	62.0 (58.0–65.2)	61.0 (58.0–66.0)	0.767
Diagnosis				0.352
STEMI	165 (20.5%)	115 (21.5%)	50 (18.7%)	
NSTEMI	145 (18.0%)	90 (16.8%)	55 (20.5%)	
Unstable angina	494 (61.4%)	331 (61.8%)	163 (60.8%)	
Procedures				
Coronary angiography	783 (97.4%)	522 (97.4%)	261 (97.4%)	1
LM	49 (6.1%)	35 (6.5%)	14 (5.2%)	0.466
LAD	597 (74.3%)	388 (72.4%)	209 (78.0%)	0.087
LCX	441 (54.9%)	281 (52.4%)	160 (59.7%)	0.051
RCA	423 (52.6%)	260 (48.5%)	163 (60.8%)	<0.001
Number of vascular lesions				0.005
0	86 (10.7%)	64 (11.9%)	22 (8.2%)	
1	220 (27.4%)	161 (30.0%)	59 (22.0%)	
≥2	498 (61.9%)	311 (58.0%)	187 (69.8%)	
PCI	507 (63.1%)	319 (59.5%)	188 (70.1%)	0.003
CABG	37 (4.6%)	28 (5.2%)	9 (3.4%)	0.234
Medications on discharge				
Aspirin	786 (97.8%)	525 (97.9%)	261 (97.4%)	0.613
P2Y12 inhibitors	733 (91.2%)	487 (90.9%)	246 (91.8%)	0.66
β-blockers	643 (80.0%)	419 (78.2%)	224 (83.6%)	0.071
ACEIs/ARBs	490 (60.9%)	325 (60.6%)	165 (61.6%)	0.798
Statins	790 (98.3%)	530 (98.9%)	260 (97.0%)	0.082

Data are presented as n, mean ± *SD*, median (interquartile range), n (%), or n/N (%), unless otherwise stated. *ACEI*, angiotensin-converting enzyme inhibitor; *ARB*, angiotensin receptor blocker; *BMIP*, body mass index; *BP*, blood pressure; *CABG*, coronaryartery bypass grafting; *HDL-C*, high-density lipoprotein cholesterol; hs-*CRP*, high-sensitivity *C*-reactive protein; *LAD*, left anterior descendingartery; *LCX*, left circumflex artery; *LDL-C*, low-density lipoprotein cholesterol; *LM*, left main artery; *LVEF*, left ventricular ejection fraction; *NSTEMI*, non-*STP*-segment-elevation myocardial infarction; *PCAD*, premature coronary artery disease; *PCI*, percutaneous coronary intervention; *RCA*, right coronary artery; *STEMI*, *ST*-segment-elevation myocardial infarction. “Number of vascular lesions = 0” indicates that coronaryangiography did not reveal any significant coronary artery stenosis or obstruction, or that the stenosis diameter was <50%

### Outcomes according to TyG group

**Fig. 2:**
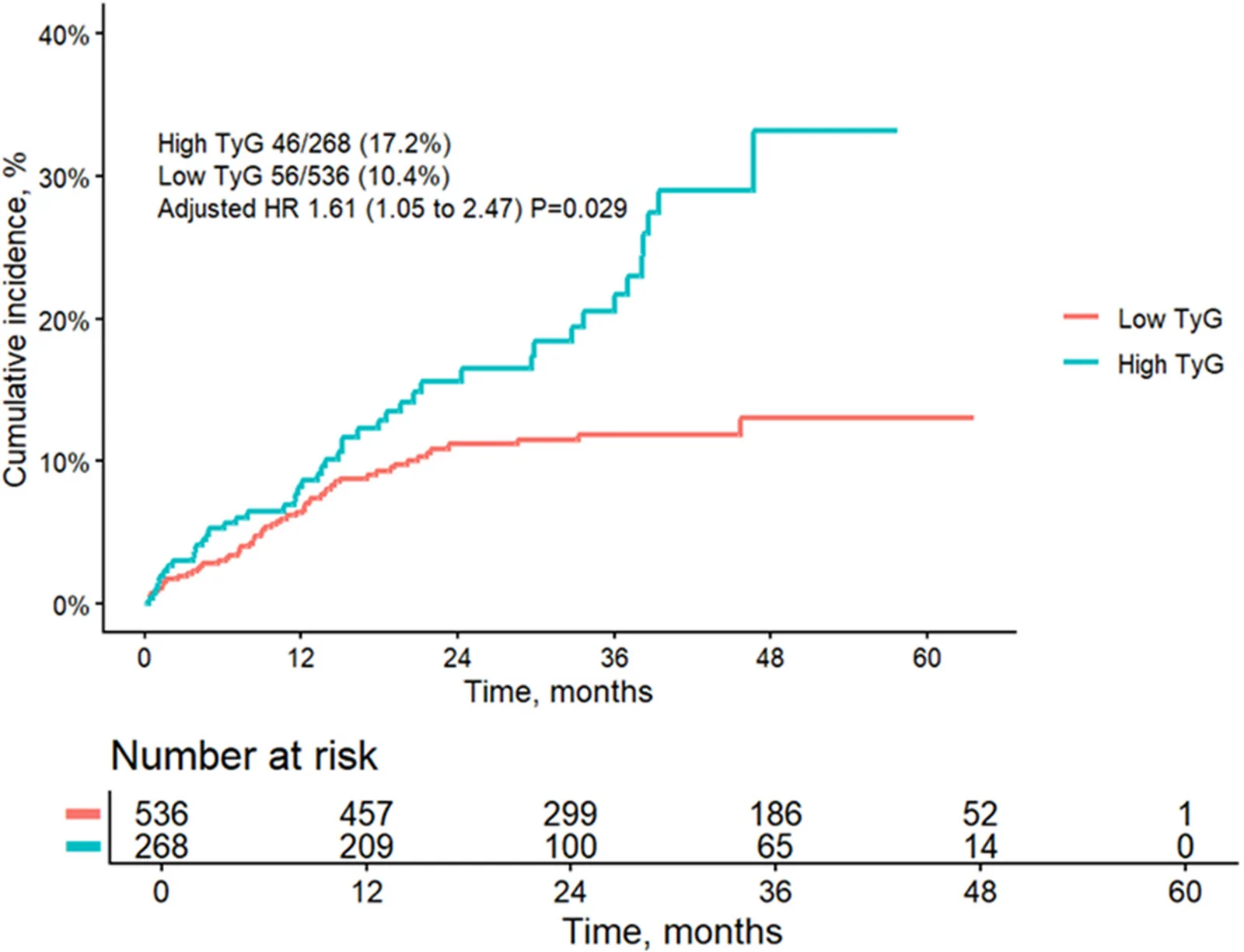
Kaplan-Meier curves for cumulative MACCE incidence by TyG group. The high TyG group showed a higher cumulative incidence of MACCE than the low TyG group during follow-up. The adjusted hazard ratio for high versus low TyG was 1.61 (95% CI, 1.05–2.47; *P* = 0.029). MACCE, major adverse cardiovascular and cerebrovascular events; TyG, triglyceride-glucose index

**Fig. 3:**
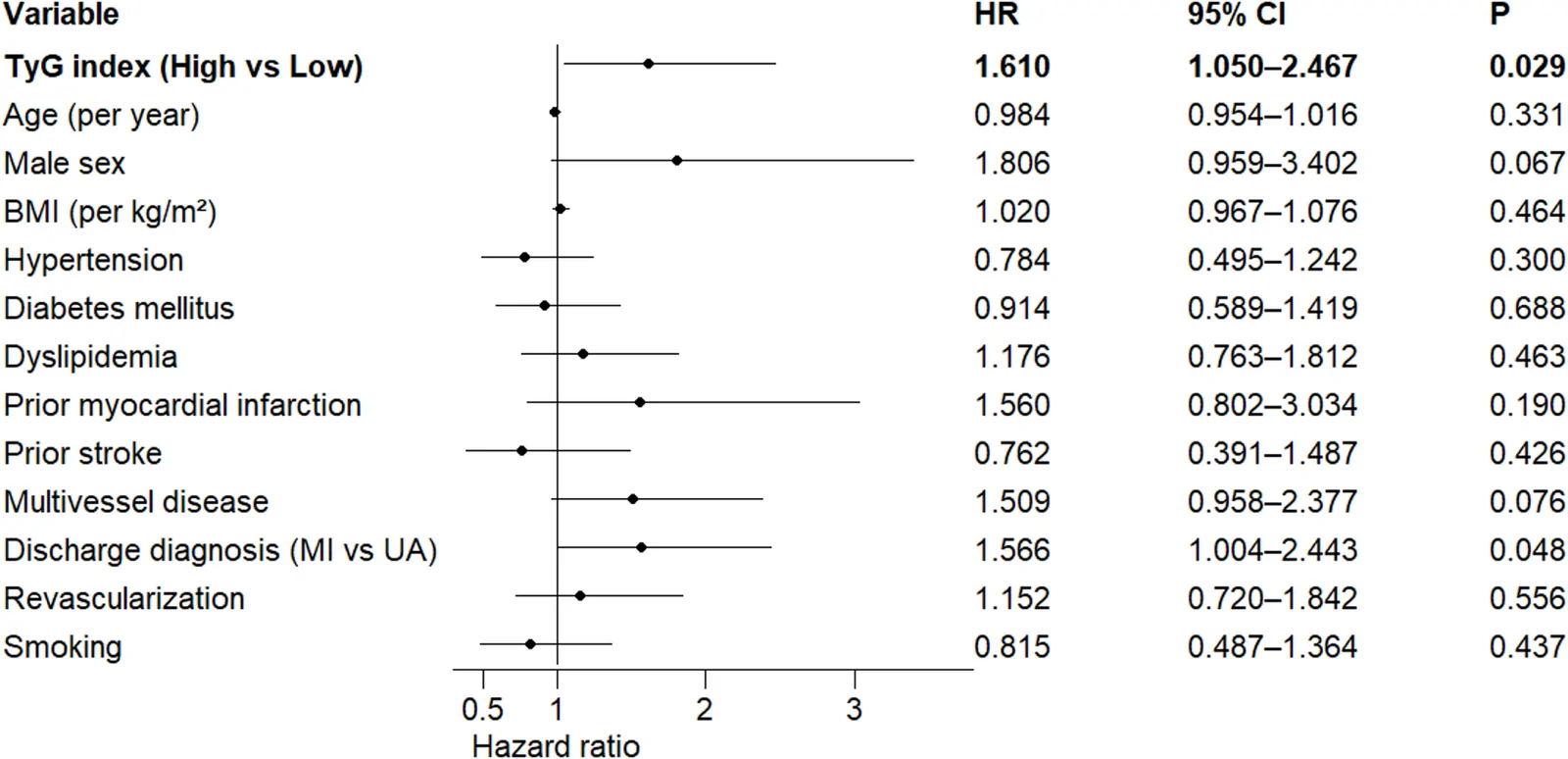
Forest plot of the fully adjusted Cox regression model for MACCE. Hazard ratios (HRs) and 95% confidence intervals (CIs) are shown for variables included in the multivariable Cox proportional hazards model. High TyG remained significantly associated with MACCE in the multivariable model. ACS, acute coronary syndrome; CI, confidence interval; HR, hazard ratio; MI, myocardial infarction; UA, unstable angina; TyG, triglyceride–glucose index

The median follow-up duration was 36 months. MACCE occurred more often in the high TyG group than in the low TyG group (17.2% vs. 10.4%) (Table 2). Kaplan-Meier curves showed a significantly higher cumulative incidence of MACCE among patients with high TyG (log-rank *P* = 0.026) (Fig. 2). In the multivariable Cox model, high TyG remained independently associated with increased MACCE risk after adjustment for conventional confounders (adjusted HR 1.61, 95% CI 1.05–2.47; *P* = 0.029) (Fig. 3).

When TyG was modeled as a standardized continuous variable, the association with MACCE persisted after multivariable adjustment (adjusted HR 1.306, 95% CI 1.039–1.643; *P* = 0.022). In a sensitivity analysis using the median TyG value as an alternative cutoff (8.954), the higher-half group showed a directionally higher incidence of MACCE than the lower-half group (14.7% vs. 10.7%). The adjusted estimate was also above unity, although it did not reach statistical significance (adjusted HR 1.30, 95% CI 0.86–1.97; *P* = 0.215). Restricted cubic spline analysis did not identify a statistically significant nonlinear pattern, suggesting that the observed association was not mainly attributable to a threshold-type nonlinear relationship.

**Table 2:**
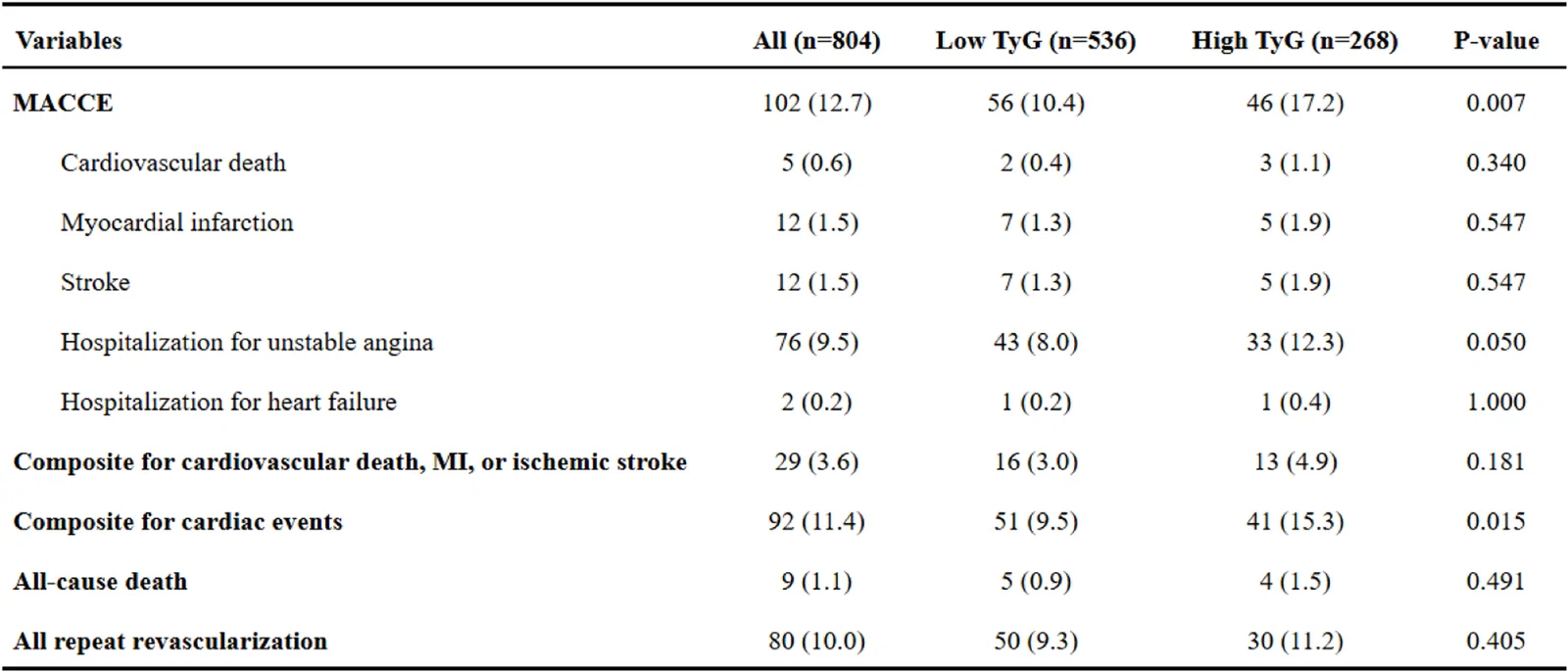
Clinical events during 3-year follow-up according to TyG group

Dataare presented as n (%), unless otherwise stated. # includes cardiovascular death, myocardial infarction, ischemia-driven revascularization, stroke, or hospitalization for unstable angina orheart failure. The multivariable Cox model was adjusted for sex, age, body mass index, smoking, hypertension, diabetes mellitus, dyslipidemia, prior myocardial infarction, prior stroke, revascularization, multivessel disease, and clinical presentation (acute myocardial infarction vs unstable angina). Univariate and multivariable Cox regression was not performed because of no events or too few events. *CI* confidence interval, *HR* hazard ratio, *MACCE* major adverse cardiovascular and cerebrovascular events

Evaluation of the proportional hazards assumption indicated that the relationship between TyG and MACCE was not constant throughout follow-up. To better characterize this temporal pattern, an exploratory landmark analysis was performed using 365 days as the landmark. Within the first 365 days after discharge, high TyG was not significantly associated with MACCE (HR 1.139, 95% CI 0.631–2.056; *P* = 0.667). Beyond 365 days, however, high TyG was associated with a significantly higher risk of MACCE (HR 2.322, 95% CI 1.228–4.388; *P* = 0.010). These results suggest that the prognostic relevance of TyG may be more evident during later follow-up.

In analyses of individual MACCE components, TyG was not significantly associated with any single endpoint (all *P* > 0.05). This finding should be interpreted cautiously because the number of events for each component was small, reducing statistical power for component-specific analyses.

## Discussion

In this retrospective cohort of patients with PACS, elevated TyG was independently associated with a higher risk of MACCE during follow-up. Patients with high TyG also had a distinctly adverse cardiometabolic profile and a greater burden of coronary artery disease at baseline. These findings suggest that, among patients who experience ACS at a premature age, TyG may reflect both underlying metabolic vulnerability and subsequent long-term cardiovascular risk.

The present results add to the growing literature on residual risk after ACS by focusing on a younger population in whom long-term prevention is especially important. Although treatment strategies for ACS have improved substantially [1], the global burden of ACS remains high [2]. Younger patients with myocardial infarction often have a high prevalence of modifiable risk factors [3], and recent evidence indicates that premature acute myocardial infarction can be followed by persistent ischemic risk, particularly in the presence of multivessel disease [4]. Because patients with PACS may live for many years after the index hospitalization, simple markers that can help identify individuals requiring sustained cardiometabolic surveillance have practical value [24].

TyG is commonly used as a surrogate of insulin resistance [5], and its correlation with clamp-derived insulin sensitivity supports its biological plausibility [6]. Mechanistically, insulin resistance and lipotoxicity may contribute to endothelial dysfunction, oxidative injury, low-grade inflammation, and atherosclerotic progression [7]. In younger patients with PACS, a higher TyG index may also indicate a longer future exposure window to insulin resistance, atherogenic dyslipidemia, low-grade inflammation, and endothelial dysfunction after the index event. This prolonged exposure may partly explain why the prognostic signal of TyG was more evident during later follow-up rather than during the early post-discharge period. Reviews of TyG in cardiovascular disease emphasize that it is best interpreted as a marker of broader cardiometabolic dysregulation rather than as an isolated lipid or glucose abnormality [8]. Consistent with this concept, recent meta-analytic evidence has connected higher TyG with coronary artery disease risk, lesion severity, and adverse prognosis [25].

Our findings are also consistent with previous studies in broader ACS and PCI populations. A dose-response meta-analysis of ACS cohorts including 13,684 patients reported that the highest TyG category was associated with an approximately twofold higher risk of MACE compared with the lowest category (RR 2.09, 95% CI 1.59–2.76; *P* < 0.001) [9]. In patients with diabetes and ACS, Wang et al. found that TyG independently predicted 3-year MACE and identified an optimal TyG cutoff of 9.323, although the discriminatory ability was modest (AUC 0.560) [10]. In ACS patients undergoing PCI, Xiong et al. reported that TyG remained independently associated with MACEs after adjustment for the GRACE score (HR 1.654, 95% CI 1.156–2.368; *P* = 0.006), and that adding TyG to the GRACE score increased the C-statistic from 0.735 to 0.744 [13]. These data support the relevance of TyG in ACS risk stratification, while the present study extends this evidence to the relatively understudied PACS population.

An important observation in this study was the time-dependent nature of the TyG-MACCE association. The association was not significant during the first 365 days but became more pronounced after the first year. This pattern may indicate that TyG is more closely related to persistent residual cardiometabolic risk than to early events driven by acute plaque instability, procedural factors, or immediate post-discharge treatment. From a statistical standpoint, when proportional hazards are not fully satisfied, the HR from a standard Cox model represents an overall average across follow-up [21]. Landmark analysis therefore provides a clinically interpretable way to separate early and later risk periods [22]. In parallel, restricted cubic spline analysis helps evaluate whether the exposure-outcome relationship is nonlinear rather than assuming a prespecified threshold effect [23]. Together, these analyses support the interpretation of TyG as a marker more relevant to long-term risk stratification after PACS. In the present cohort, discharge secondary-prevention medications were broadly comparable between the TyG groups, suggesting that the delayed separation of event curves was unlikely to be explained solely by differences in discharge treatment intensity. However, detailed information on long-term medication adherence, post-discharge glucose and lipid control, lifestyle modification, and cardiac rehabilitation participation was unavailable. Therefore, the observed late risk separation should be interpreted as hypothesis-generating rather than definitive evidence of a specific causal mechanism.

From a clinical perspective, TyG has several pragmatic advantages: it is inexpensive, derived from routinely measured fasting triglyceride and glucose values, and, unlike conventional insulin-based indices such as HOMA-IR, does not require fasting insulin testing. Recent AMI studies also suggest that TyG is associated with long-term adverse cardiovascular outcomes after PCI [26] and with intracoronary thrombus burden in patients with STEMI undergoing primary PCI [27]. It may therefore serve as an adjunct for identifying PACS patients who could benefit from closer follow-up and more intensive long-term management of metabolic risk. However, TyG should not be viewed as replacing established clinical risk assessment or guideline-directed secondary prevention after ACS [20]. Instead, it may help highlight a residual cardiometabolic risk dimension that is not fully captured by traditional variables alone.

## Limitations

First, the study was retrospective and conducted at a single center; therefore, selection bias and residual confounding cannot be excluded despite multivariable adjustment. Second, TyG was measured only at baseline. Dynamic changes in triglycerides, glucose, diabetes treatment, lipid-lowering therapy, and lifestyle modification during follow-up were not captured, although prior evidence suggests that longitudinal TyG patterns may add prognostic information [14]. Third, information on adherence to secondary prevention and post-discharge risk factor control was limited, which may have influenced long-term outcomes. Fourth, the number of individual component events was small, limiting the ability to determine which MACCE component contributed most strongly to the observed association. Fifth, the landmark and spline analyses should be regarded as exploratory and require confirmation in larger prospective cohorts. In addition, fasting insulin data were missing in a substantial proportion of patients, which precluded reliable calculation of HOMA-IR and prevented direct comparison of the prognostic performance of different insulin resistance indices in this PACS cohort. Finally, although the results support an association between TyG and prognosis in PACS, they do not establish causality, and further multicenter studies are needed to clarify the clinical role and optimal use of TyG in this population [8].

## Conclusion

In patients with premature acute coronary syndrome, elevated TyG was independently associated with a higher risk of MACCE during follow-up. This association appeared stronger beyond the first year after discharge, suggesting that TyG may help identify long-term residual cardiometabolic risk in this relatively young high-risk population. Prospective multicenter studies are needed to validate the temporal risk pattern and to determine how TyG may be incorporated into post-PACS risk stratification and follow-up management.

## Data Availability

The datasets analysed during the current study are not publicly available due to institutional and patient privacy restrictions, but are available from the corresponding author on reasonable request and with appropriate institutional approval.
